# Physical Activity and Post-Transcriptional Regulation of Aging Decay: Modulation of Pathways in Postmenopausal Osteoporosis

**DOI:** 10.3390/medicina58060767

**Published:** 2022-06-06

**Authors:** Federica Vita, Sebastiano Gangemi, Giovanni Pioggia, Fabio Trimarchi, Debora Di Mauro

**Affiliations:** 1 Unit and School of Allergy and Clinical Immunology, Department of Clinical and Experimental Medicine, University of Messina, 98125 Messina, Italy; federica.vita75@gmail.com (F.V.); sebastiano.gangemi@unime.it (S.G.); 2 Institute for Biomedical Research and Innovation (IRIB), National Research Council of Italy (CNR), 98164 Messina, Italy; giovanni.pioggia@cnr.it; 3 Department of Biomedical Sciences, Dental Sciences and Morpho-Functional Imaging, University of Messina, AOU “G. Martino”, 98125 Messina, Italy; fatrim@unime.it

**Keywords:** osteoporosis, physical activity, miRNAs, training, irisin, high-intensity interval exercise, continuous moderate-intensity exercise, resistance exercise

## Abstract

*Background and Objectives*: Bones and the skeletal muscle play a key role in human physiology as regulators of metabolism in the whole organism. Bone tissue is identified as a complex and dynamic living unit that could react to physical activity. Hormones, growth factors, signaling factors, and environmental factors control osteogenesis, and it could be regulated at a post-transcriptional level. MicroRNAs (miRNAs) can interfere with mRNAs translation. Increasing data suggest that miRNAs, through different pathways, are involved in the regulation of bone marrow mesenchymal stem cells (BMSCs) differentiation and physical activity-induced bone remodeling. The purpose of this narrative review is to investigate the potential protective role played by physical activity in affecting miRNAs expression in close tissues and elaborate on the complex network of interplay that could drive various metabolic responses of the bone to physical activity. *Materials and Methods:* A bibliographic search of the scientific literature was carried out in scientific databases to investigate the possible effect of physical activity on age-related features detected in the musculoskeletal system. *Results*: Several studies suggested that the musculoskeletal system interacting at a biomolecular level could establish crosstalk between bone and muscle in an endocrine or paracrine way through myokines released by muscle at the periosteal interface or in the bloodstream, such as irisin. Mechanical stimuli have a key role in bone formation and resorption, increasing osteogenesis and downregulating adipogenesis of BMSC via regulation of expression of runt-related transcription factor 2 (Runx2) and peroxisome proliferator-activated receptor gamma (PPARγ), respectively. *Conclusions:* Increasing data suggest that miRNAs, through different pathways, are involved in the regulation of BMSCs differentiation and physical activity-induced bone remodeling. Modulation of miRNAs following physical exercise represents an interesting field of investigation since these non-coding RNAs may be considered defenders against degenerative diseases and as well as useful prognostic markers in skeletal and muscle-skeletal diseases, such as osteoporosis.

## 1. Introduction

The bone and the skeletal muscle play a key role in human physiology since they not only fulfill a mechanical role in locomotion and movement but are also prominent regulators of metabolism in the whole organism. Bone tissue is identified as a complex and dynamic living unit that could react to physical activity or any type of mechanical loading such as exercise training [1]. A recent work suggested that the musculoskeletal system interacts at a biomolecular level, establishing that crosstalk between bone and muscle takes place in an endocrine or paracrine way through myokines released by muscle at the periosteal interface or in the bloodstream. Among these molecules, a central role is played by the newly recognized myokine, irisin, that acts in the muscle-bone unit, with a greater impact on the bone [2]. Irisin is a peptide hormone of 112 amino acids that was illustrated as a myokine produced from fibronectin type III domain-containing5 (FNDC5) and proteolytic division that support white adipose tissue (WAT) browning [3]. 

Bone mass constitutes approximately 14% and 11% of total body mass in females and males. Moreover, mechanisms of age-related acceleration in bone loss in older males are different from those associated with menopause. Age-related reduction in the osteogenic differentiation has been connected to adipo-osteogenic differentiation of bone marrow mesenchymal stem cells (BMSCs) [4]. It is well-known that mechanical stimuli can highly influence skeletal homeostasis and BMSCs and seems to have a key role in bone formation and resorption, increasing osteogenesis and downregulating adipogenesis of BMSC. Mechanical loading could influence the differentiation of mechanosensitive mesenchymal stem cells into osteoblast or preadipocytes through the regulation of some transcription factors, such as runt-related transcription factor 2 (runx2) [5] and expression of peroxisome proliferator-activated receptor gamma (PPARγ) [6]. In addition to being controlled by hormones, growth factors, signaling factors, and environmental factors, osteogenesis is also regulated at a post-transcriptional level. MicroRNAs (miRNAs) can interfere with their mRNA translation. Increasing data suggest that miRNAs, through different pathways, are involved in the regulation of BMSCs differentiation and physical activity-induced bone remodeling [7]. Therefore, PPARγ overexpression in marrow stromal cells from aging people might define the role of some miRNAs in this pathway and their possible involvement in controlling the same signal network also in estrogen deficiency-induced bone loss [8].

The literature data show that regular physical activity is one of the most widely used nonpharmacological strategies that help to improve human performance status and can help to obstruct several diseases such as age-related muscle wasting, obesity, and osteoporosis (OP) [9]. Modulation of miRNAs following physical exercise could represent an interesting field of investigation since these non-coding RNAs might be considered defenders against degenerative diseases, as well as useful prognostic markers [7].

OP is a set of alterations that include reduction in bone quality and density, impairment of skeleton, and consequently increment of risk of fracture [10]. The excessive decay could be caused by a series of factors, including disuse, and immobility may also concur to the pathogenesis of OP [11]. In general, it has been proved that bone reacts to mechanical strain in an intensity-dependent manner, and the OP prevention training scheme has typically advised moderate-intensity exercise [12]. 

In the literature, there are not enough accurate studies that compare different types of intensity training (resistance and endurance training) plans and estimate miRNAs variable expression in adipo-osteogenic trans differentiation of BMSCs [12]; but, there is more evidence that claimed miRNAs such as diagnostic, prognostic and/or predictive resources in skeletal and muscle-skeletal disease, such as OP [13]. The still restricted data about the role of miRNAs in OP is mainly elicited from different numbers and types of human samples, including serum, bone tissue, BM-MCSs, or circulating monocytes. Moreover, these findings were derived mainly from patients of distinct ethnicities, some with bone fractures or low bone mineral density (BMD), and compared with healthy controls or patients suffering from other pathologies [14].

The purpose of this narrative review is to investigate the potential protective role played by physical activity in affecting miRNAs expression in close tissues and elaborate on the complex network of interplay that could drive various metabolic responses of the bone to physical activity. Thus, it could lead to the establishment of peculiar positive crosstalk in the skeleton-muscle system. 

## 2. Materials and Methods

A bibliographic search of the scientific literature was carried out in scientific databases to investigate the possible effect of physical activity on age-related features detected in the musculoskeletal system. Consequently has been evaluated if regulation of osteogenic differentiation, through changes in the production of transcription factors or modulation of miRNAs typical of postmenopausal osteoporosis, could lead to a possible improvement in disease. The MeSH terms “microRNAs” and “Osteoporosis” were used in association with “Physical activity”. The search strategy related to the population, intervention, and outcomes was constructed around search terms for “Bone Mineral Density”, “osteogenesis”, “Exercise,” “high-intensity interval training”, “resistance exercise”, “endurance”, “continuous moderate-intensity exercise”, “modulation miRNAs” and “Postmenopausal”. The present study was based on comprehensive reviews and research studies on both mice models and human samples. The research was conducted by findings that led to an improvement in osteoporotic status. The criteria of inclusion in the study were considered the factors that after different physical activity helped to improve musculoskeletal system aging at different biomolecular levels on various linked signaling pathways.

## 3. Fate of MSCs: Age-Related Alterations

Both patients with postmenopausal osteoporosis [15,16] and ovariectomized (OVX) rats showed an increase in adipocytes in bone marrow parameters associated with an inverse link between the trabecular bone density and the total of bone marrow fat tissue [17]. Bone health has been demonstrated by the mice knockout (KO) model for brown adipose tissue (BAT), highlighting an increase in bone resorption and impaired bone formation [3]. 

**Figure 1:**
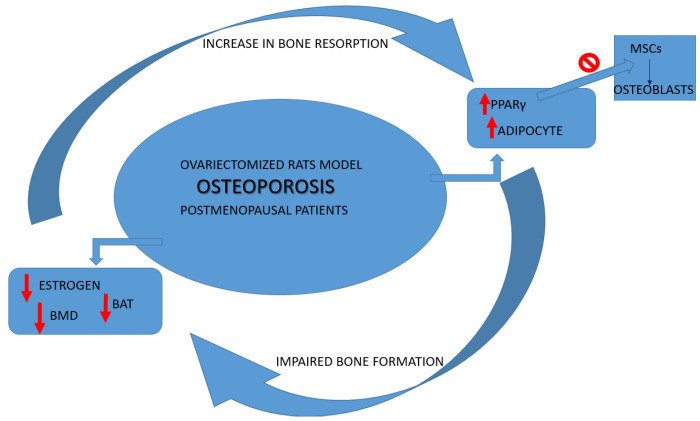
Osteoporosis cycle alterations. BAT: adipose tissue browning; PPARγ: proliferator-activated receptor gamma; BMD: bone mineral density; MSCs: marrow mesenchymal stem cells.

It has also been shown that PPARγ influences bone loss in humans and animals partly through the limitation of osteoblast differentiation from BMSCs [18]. According to the model of OVX-induced osteoporosis, it has been shown that, respectively, the amount of protein levels of PPARγ and fat vacuoles was increased, and the femur and vertebrate BMD and estrogens serum levels were decreased [19]. To better define the direct effects of PPARγ on osteoblasts, Cho SW et al. in 2011 showed in vivo on female mice that osteoblast-specific overexpression of PPARγ, obtained using collagen type 1 promoter, accelerated estrogen-deficiency-related bone loss, suggesting a possible role of estrogens in regulating PPARγ pathways [20] (Figure 1).

### Fat Tissue and Bone-Muscle Unit: Physical Activity and Irisin, Myostatin, Sclerostin Crosstalk

Endurance activity and muscle-specific overexpression of peroxisome proliferator-activated receptor gamma coactivator (PGC)—1 alfa stimulates FNDC5 expression in skeletal muscle and consequently leads to increased irisin level [3]. Irisin, compared to other myokins, is more sensitive to different types of physical activity. It raises more in people following resistance training and high-intensity exercise than after endurance exercise. Irisin produced by physical activity has a potential virtuous effect on inducing adipose tissue browning (i-BAT). This recent anabolic finding of BAT develops through the downregulation of sclerostin gene expression on osteocytes and the increasing of phosphor-Akt and B-catenin expression on osteoblastic cells. Therefore irisin, suppressing the RANKL-Akt1/MITF/PU1-NFATc1 pathway, inhibit osteoclast differentiation thanks to the stimulation of bone morphogenic proteins (BMP) gained by osteoblast differentiation [21,22].

Age-related changes in irisin levels are less marked in men compared to women; in fact, Park et al. in 2019 reported in postmenopausal women that sarcopenia status was characterized by low levels of circulating irisin [23]. Previously evidence found that irisin serum levels, although it was independent of BMD, were negatively associated with both vertebral brittle fractures in postmenopausal women [24] and serum sclerostin levels, a protein expressed by osteocytes and inhibitor of bone formation. The study of Yan J. et al. in 2018, on the contrary, was the first where 160 older women with minimal trauma hip fracture compared to control without fracture showed an increased risk of minimal trauma hip fractures at low irisin serum levels. Indeed they underlined the new link between serum irisin and bone mass density and the negative influence of age related to irisin serum levels [11]. In 2019, Zhou K. et al. conducted the first meta-analysis to better define the role of circulating irisin levels in older and middle-aged osteoporotic patients. This analysis included five studies about postmenopausal women and two about both elderly women and men; it corroborated the opportunity to be able to use irisin as biomarkers for following the OP course [25]. The aforementioned findings induced by OVX were improved after physical activity and might be normalized by estrogen replacement. Moreover, exercise has been demonstrated to be a beneficial effect mostly on cancellous bone, the type of bone that is more affected by the loss induced by OVX. Chen Y. et al., in 2011, were the first ones to show in the mice OVX model that PPARγ levels in bone samples might be influenced more by mechanical loading than estrogen replacement. Differently from previous studies that demonstrated, both in vitro and in vivo, the promotion impact of physical activity on osteogenic factor Runx2 [5,26], Chen et al. showed that moderate exercise has a greater influence on inhibition of adipogenesis factor rather than osteogenic factor Runx2 [19].

## 4. Influence of Physical Activity on Post-Transcriptional Regulation of Fat, Muscle, and Bone Tissue Pathways

**Figure 2:**
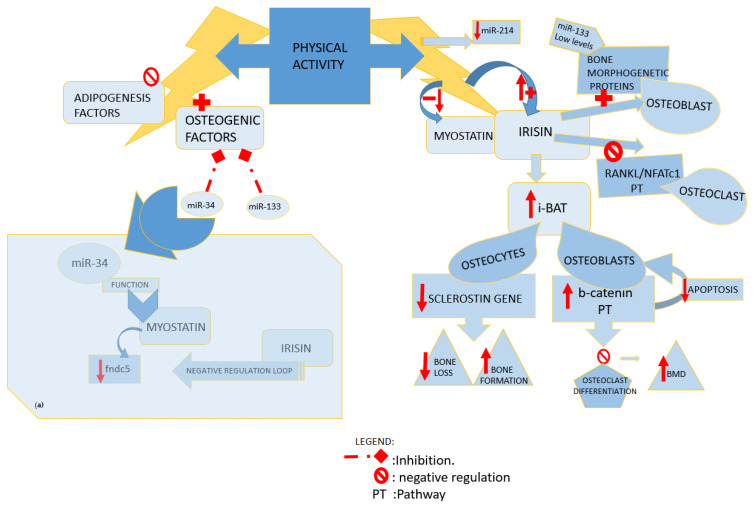
Influence of physical activity. (**a**) miR34 function. miR: microRNA; fndc5: fibronectin type III domain-containing protein 5; RANKL: receptor activator of NFκB ligand; NFATc1: nuclear factor of activated T cells; i- BAT: induced adipose tissue browning; BMD: bone mineral density; PT: pathway.

Data showed that irisin and its precursor were overexpressed in skeletal muscle of myostatin KO mice line, underlining a signaling pathway activated by mutation of myostatin that, indirectly, drives browning of WAT through the regulation of its pathway (AMPK-PGC1-Fndc5) [27,28]. A more recent study investigated on post-transcriptional regulation of myostatin, and the authors showed that myostatin downregulates Fndc5 through the miR-34 pathway in both white adipocytes and myoblasts. Even if they showed that myostatin upregulates miR-34, the molecular mechanism behind this pathway remains to be investigated [29]. Ge et al., differently from Shan et al., found down-expression of fndc5 mRNA, together with the upregulation of fndc5 protein levels in skeletal muscle of myostatin KO mice. This suggested the potential existence of a negative autoregulatory feedback loop, where the raised fndc5/irisin protein levels found in muscle and in circulation, in turn, inhibit fndc5 expression. Both irisin and myostatin proteins are well conserved across species, with identical amino acid sequences of mature irisin and myostatin between humans and mice [3]. Given the fact that also human and mouse miR-34 are conserved across species, it is possible that the same pathway can be replicated in humans [29] (Figure 2(a)).

The levels of biomarkers (runx2, alkaline phosphatase, osteocalcin, osterix), known as osteogenic markers, change following the various stages of osteoblast differentiation; however, in order to modulate monocyte to osteoclast differentiation, osteoblasts may produce receptor activator of NF-kB ligand (RANKL) and osteoprogeterin (OPG) such as macrophage colony-stimulating factor (M-SCF). RANKL/RANK, jagged1/Notch1, and Wnt/b-catenin are three significant regulatory feedbacks driven by osteoblasts, which could affect the BMD through the adjustment of osteoclast and osteoblast functions. Various studies investigated the modulation of these pathways and also showed a gain of progenitor cell number in peripheral blood after different physical exercises [30]. In 2019, Dalle Carbonare L. et al. confirmed the key role of physical activity in inducing osteogenic differentiation [31]. Yet, in 2017 Singulani et al. had shown a positive modulation of bone biomechanical parameters by in vivo and in vitro studies in aging female mice models through modulating processes such as adipocytes differentiation and incitement of osteoblast differentiation [32].

Osteogenesis could be induced through the irisin naturally produced after exercise training as well as by the recombinant irisin (r-irisin) injected [33]. Colaianni et al. in 2015 showed that irisin myokine enhanced the markers of the early and late phases of osteoblastogenesis, respectively, alkaline phosphatase (ALP) and an array of mineralized nodules, through the upregulation of osteoblast differentiation transcription factors (RUNX2 Atf4 and osterix) mRNA levels and the start-up of Wnt pathway [34]. R-irisin treatment produced in vivo a sclerostin-related bone reaction to mechanical unloading through the weakening of the Wnt/beta-catenin pathway, which causes a decrease in sclerostin mRNA expression. R-irisin, in vitro, led to an increment of nuclear localization of beta-catenin on osteoblast precursor cells of mice models [33], and it inhibited RANKL-induced osteoclast differentiation resulting in improvements in microstructure and BMD [35]. Indeed, bone mass is sustained by sclerostin through inhibition of the Wnt/b-catenin pathway. Wnt pathway influences in a negative and positive manner, respectively, mature osteoblast apoptosis and stimulation of osteoprogenitor [30] (Figure 2).

Previously data have been already shown an upregulation of irisin blood levels after swimming exercise, but Kang et al. also reported a direct impact on BMD operating in a positive manner: indirectly on IL-1 and body fat reduction and directly on bone metabolism marker pathways such as beta-catenin, osteocalcin, and CTX-1, increasing PGC-1alfa/FNDC5 expression in bone tissue [35]. Other findings showed that high-intensity interval training (HIIT), relative to the moderate-intensity continuous exercise (MICE) training, may elevate irisin levels rates to a greater extent, it may increase fat oxidation during and following exercise, and it may recruit more type II muscle fibers, which have a better oxidative capacity using more lipids and less glycogen. Moreover, a high-intensity exercise training, in spite of its short duration, can enhance the mitochondrial capacity/biogenesis of skeletal muscle, leading to an increase in PGC-1α mRNA three hours following acute HIIT [36]. Liu et al. in 2021 underlined how high-intensity interval static training prevents skeletal muscle atrophy and improves the motor function of aged rats through the PGC-1α/FNDC5/UCP1 pathway [37]. Eaton et al. in 2018 conducted the first study that examined the effects of HIIT on FNDC5 mRNA expression. These findings indicate that resting FNDC5 mRNA levels in human muscle are responsive to short-term high-volume periods of HIIT and may represent an adaptive skeletal muscle response to HIIT. Following 20 days of high-volume twice-daily HIIT, the same acute bout of HIIE increased IL-6 and FNDC5 as compared to resting levels, and overall FNDC5 mRNA expression was increased after training [38] (Table 1).

### 4.1. Impact of Different Types of Exercise on Bone Metabolism

Turner et al. showed that the osteogenic reaction to mechanical stimuli was greater in young rats compared to aged ones, even if, once activated, the cells of older rats had the similar power to reply to mechanical loading as the young ones [39]. A recent study on risk factors and complaints related to the menopausal transition or increasing age was conducted after 18 months of multipurpose exercise, including aerobic dance with moderate to high ground reaction forces, jumping and resistance. It has been reported a less effect on BMD as compared to older people investigated in a previous study, and it could probably be conjectured that the different effects of different programs were caused by the early-postmenopausal status of this sample [40].

**Table 1:** Physical activity and modulation pathways.

Reference Year Author	Article Type	Type of Sample and Analysis	Physical Activity Type and Duration	Pathways Involvement	Effects
[31] 2019 Dalle Carbonare L. et al.	Article	-H.C. -n.22 M healthy and regularly active -Vitro analyses before and post run	-Half marathon performance	Upregulation of osteogenesis related genes Downregulation of Adipogenic commitment	Post activity increases sera levels of: RUNX2, MSX1, SPP1, BMP2, BMP6, and PPARγ gene Exercise counteracts chronic degenerative conditions
[30] 2020 Tobeiha, M. et al.	Review 21 studies	In 4 studies -O, F; -H/A	In most of the studies: high-intensity exercise	RANKL/RANK/ OPG pathway	Increases level of: -OPG Decreases level of: -RANKL Exercise promotes bone health
[35] 2019 Kang, Y.S. et al.	Article	-n.20 A.	-Swimming exercise. -16 weeks study period	Bone metabolism marker pathways beta-catenin	Increases level of: -Serum irisin - Bone tissue PGC-1α and FNDC5
[36] 2015 Kim, H.J. et al.	Control study	-n.28 obese H, M/F -Y -3 h following acute HIIT	-HIIT vs. MICE. -8 weeks of exercise program (60 min/day, 5 times a week)		HIIT increases: -Fat oxidation during and following exercise -PGC-1α mRNA -Serum irisin level
[37] 2021 Liu, Y. et al.	Control study	-n.40 O, A.	-High-intensity interval static training -8 weeks	Regulation of PGC1α/FNDC5/UCP1 signaling pathway	Increased levels of: -Serum irisin improvement in motor function of aged rats
[38] 2017 Eaton, M. et al.	Article	-n.10 H, Y, M. -Valuation before and after activity	-HIIT -20-day period of twice-daily	acute regulation of the mRNA myokine, interleukin-6, and FNDC5	Increase in: -FNDC5 mRNA expression
[41] 2018 H. Shirvani and Arabzadeh, E.	Article	-n.32 A, M.	-HIIT vs. MICE -8 weeks	Crosstalk between skeletal muscle and adipose tissue	Increase in: -PGC-1α gene transcription; -Serum irisin

Legend Table 1: S: sex; H: human; A: animal test (mice or rats); M: male; F: female; O: old; Y: young; C: cells. HIIT: high-intensity interval training; MICE: moderate-intensity continuous exercise; OPG: osteoprogeterin, RANK: receptor activator of NFκB; RANKL: receptor activator of NFκB ligand; RUNX2: runt-related transcription factor 2; MSX1: Msh homeobox 1; SPP1: secreted phosphoprotein 1; BMP2: bone morphogenetic protein2; BMP6: bone morphogenetic protein6; PPARγ: proliferator-activated receptor gamma; PGC-1 alfa: peroxisome proliferator-activated receptor gamma coactivator-1alfa; FNDC5: fibronectin type III domain-containing protein 5 gene; UCP1: uncoupling protein 1.

Many studies have shown that long-term aerobic exercise enhances PGC-1α expression levels in the skeletal muscles of high-fat diet-fed mice, resulting in the aerobic oxidation of fatty acids. However, this physical activity is inconvenient for older people while considering static strength training (ST) mode more suitable for older people [37]. A positive influence on the regulation of expression of PGC-1α and crosstalk of signals between the skeletal muscle and adipose tissue has been shown through two HIIT and moderate-intensity continuous training (MICT) exercise modalities following 8 weeks [41]. To obtain better results for the elderly, physical activity should persist for a minimum of 6 months with no break, and a 12-month clinical trial study investigated the dose-response effects of exercise with weight-bearing components on total bone mineral density compared to postmenopausal idle women. One group followed high rates of aerobic exercise, and the other was submitted to a moderate rate of aerobic exercise, showing a greater improvement in BMD in the first one; these results have been held for almost a year after the interruption of the study [42].

Weight training and weight-bearing training, such as running and walking, have been practiced to improve bone metabolism in aging [43]; moreover, other recent studies have shown that non-weight-bearing exercises such as swimming are just as valid to improve bone metabolism, focusing on BMD and bone microstructure, also in OVX rats [44,45]. It has been investigated that also high-intensity resistance and endurance exercise are able to improve bone metabolism [46,47]. Other studies, in vivo and in vitro, showed that a 16-week strength program could improve bone biomechanical characteristics via induction of osteoblast differentiation during senescent [32]. In 2008 Gudendi et al. showed that the relatively lower duration of aerobic exercise (4 weeks, twice a week) program than previous studies significantly ameliorated balance scores in postmenopausal women when compared with the control group [48]. The literature on the effect of treadmill training on murine bone shows mixed findings; recently, it has been detected that none of the three different intensity treadmill training regimens prevented OVX-induced bone loss, nor did they improve the biomechanical properties of the bone [49].

### 4.2. Involvement of Different Signaling Pathways and Related miRNAs in Bone Loss

Bone homeostasis needs a fine regulation of transcription factor gene that is either highly expressed or osteoblastic specific in cells of the osteoblast commitment lineage. Other level of regulation could include both nuclear protein such as STAT1, that could weaken the function of osterix, runx2, and or ATF4, and proteins of matrix nuclear, such as SATB2 favoring osteoblast differentiation, raising the activity of runx2.

Moreover, a superior and issued regulator key of gene expression has been represented by microRNA [50]. Along with the wnt cascade, which induces the expression of osteoprogeterin, BMPs could also be able to commit MSCs to osteoblast lineage, activating runx2 and osterix during osteoblastogenesis [51]. Indeed, miR-133 low levels have been detected during the osteoblastic differentiation in mesenchymal mice line cells induced by BMP-2; BMD alteration is typical in the OVX mice model; therefore, a meaningful increased level of miR-133 was in line with these findings in the literature data [8]. MiR-133 was also previously identified in murine models as an inhibitor of osteoblast differentiation by targeting RUNX2 [13], and miR-34 was identified among the first miRNA spiked during BMP2-mediated osteoblast differentiation. Wei et al. in 2012 showed that miR-34 inhibited osteoblast differentiation through its proliferation and downregulation of SATB2 [50]. Moreover, it has been demonstrated that miR-34 played a pivotal role in balancing the remodeling of bones also by targeting notch pathways and affecting, thus, both osteoclast and osteoblast in vivo. This overview was the result of gain- or loss-of-function experiments during osteoblast differentiation. In the early stage, a gain of notch function showed a lower bone mass and osteoblastic inhibition. In contrast, a gain of these pathway functions or a deficiency in committed osteoblastic cells respectively inhibited terminal osteoblastic differentiation leading to an osteosclerotic phenotype and downregulated OPG stimulating osteoclastogenesis, which causes, thus, age-related osteoporosis [52]. The alteration in bone metabolism investigated via overexpression of miR-34 in line mice emulated the model of loss of notch function [53]. The bone homeostasis is guaranteed through matching regulation negative feedback system; indeed, activated osteoblasts produce both RANKL and OPG; in the former one, it may act, inducing osteoclastogenesis, while in the latter, it antagonizes the osteoclastogenesis. High levels of miR-34 have been detected during the induction of osteoblast differentiation promoted by stimulation of wnt signaling in C2C1; moreover, during preosteoblast maturation, miR-34 modulates the expression of its markers: ALP and osteocalcin. As previously demonstrated by Wei et al., lack of miR-34 in osteoblast line mice leads to the upregulation of osteocalcin levels [54].

**Figure 3:**
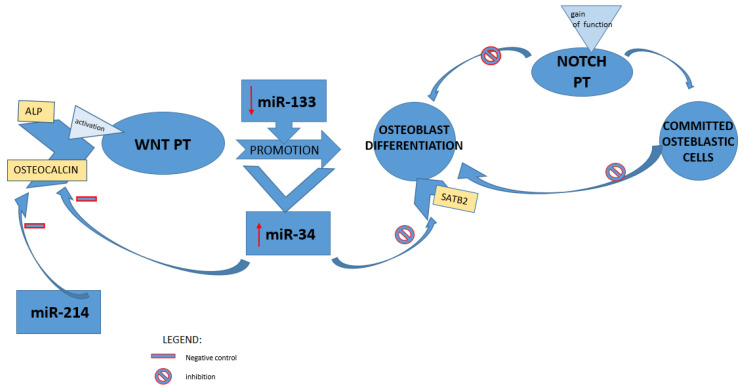
miRNAs and regulation of bone formation pathways. miR: microRNA; ALP: alkaline phosphatase; WNT PT: Wnt/β-catenin pathway; NOTCH PT: Notch signaling pathway; SATB2: special AT-rich sequence binding protein 2.

The bone expression of MiR-214 was identified as a negative regulator of the bone formation marker gene (osteocalcin and ALP) in aged people [55] (Figure 3). Consequently, miR-214 was found downregulated in both osteoblasts after mechanical strain in vitro and in tibia mice models after exercise in vivo [56].

### 4.3. Physical Activity and miRNAs Modulation in Prevent of Bone Loss: A Prospective Therapeutic Target

Mechanical strain targeting sclerostin, an inhibitor of wnt/beta-catenin pathways, might both increase osteoblastic bone formation and drop osteoclastic bone resorption [57], and it was downregulated in tibiae of irisin-treated mice [34]. Indeed, it has been shown that irisin, the counterpart of sclerostin, plays a key role in arising the osteogenic differentiation of bone marrow mesenchymal cells and in the proliferation of osteoblast mice line suppressing osteoclast formation. Moreover, to validate the beneficial effects of exercise, it has been demonstrated that a sample from myoblast, achieved from exercised muscle, was able to raise the amount of ALP colonies in the culture of undifferentiated bone marrow mesenchymal cells, and this effect was potentially reversible in the presence of antibodies against irisin in vitro. Despite in vivo data suggesting that r-irisin, by targeting sclerostin, takes part in the signal transcriptional cascade triggered by exercise, its direct effect on osteocytes is still under investigation [58,59]. In 2015, Colaianni et al. showed that r-irisin expanded osteoblast differentiation, established by nodules of mineralization matrix and ALP colonies improvement and overexpressed mRNA levels of runx2 and osterix together with wnt signal related gene b-catenin [34]. Yuan et al. showed that physical exercise could inhibit some miRNAs in bones, such as miR-214, evolutionarily conserved through many species. To confirm these findings, the upregulation of miR-214, which has been demonstrated to cause downregulation of beta-catenin and ATF4, the inhibition of ALP activity, and the increase in RANK, was detected in osteoblasts [56]. Moreover, it has been shown that pulsed systemic administration of antagomir-214 in estrogen-depleted mice restored bone mass since age-related bone loss is mainly characterized via a decrease in bone formation rather than bone loss [55].

Moreover, data showed that also miR-133 was particularly deregulated in the bone tissue of OVX mice [8] as in the serum of postmenopausal osteoporotic women [8,60]. MiR-133 filled a key role in osteoclastogenesis and has been detected inversely related to lumbar spine BMD; in addition, in vitro experiments upregulation of miR-133 during osteoclastogenesis advanced RANKL-induced differentiation of cell model line into osteoclast; raising the expression of c-Fos, NFATc1 in RANKl-treated human and murine cells line [60]. A recent study on OVX mice, in line with previous results on similar model mice [61,62] and postmenopausal women [63], validates the protective effects of exercise against bone metabolism alteration induced by estrogen on model mice. Kawao et al. in 2021 demonstrated that treadmill activity for 8 weeks mitigates the reduction in trabecular BMD induced in these mice models, and it could obstruct postmenopausal osteoporosis through elevation of irisin expression [57].

It was yet reported that WAT produced FNDC5/irisin after physical activity [64], and the marked transcription of typical BAT-like adipocytes gene, showed in myostatin KO mice, underlined the power to sustain an anabolic effect on the bones [65]. Therefore, myostatin deficiency could drive a rise in the browning of WAT, and irisin can act on WAT not only in an endocrine way but also in an autocrine manner. The loss of myostatin demonstrated by Ge, in fact, established a miR-34 downregulation, which promotes increased fndc/irisin expression and secretion [29].

Moreover, some miRNAs, miR-1-3p, miR-133a-3p, miR-133b-3p, and miR-486-5p, in whole blood from men decreased more after a 6-week short-term sprint interval training (SIT) intervention than after a single session of SIT. High-intensity exercise may reduce age-related decline factors [66] through the inhibition of myostatin and the activation of SIRTs, suggesting that mRNA targets of the miRNAs altered by SIT are linked to aging and cellular senescence (SIRT1/3, TERT, and TINF2). In addition, the alternation between low and high intensities within a single exercise bout group enhanced the expression of SIRTs, runx2, and BMPs through the inhibition of osteoclast-specific genes in aging-induced OVX rats. Therefore, this exercise mode may be recommended as a practical intervention to prevent skeletal muscle wasting and bone loss in the elderly [67].

Alipour et al. investigated the decrease in miR-34 in response to physical exercise; they showed for the first time the negative effect of swimming exercise on the expression of miR-34 on pancreatic tissue of mice with type 2 diabetes; in general, these findings displayed the modulation through physical activity on molecular axis miR-34/SIRT1/P53 [68]. Previous studies demonstrated that SIRT1 increased in response to physical activity, causing protective anti-apoptotic markers. It has been yet detected that swimming increased SIRT1 levels in soleus and gastrocnemius muscle of mice [69]. Moreover, the role of p53 levels on serum of osteoporosis patients has been investigated; it has been found that increased and knocking down p53 might reverse decreases in BMD in vivo and in vitro. Therefore, suppressing some of the functions of p53 may forbid the evolution and/or development of OP. For that reason, it could be useful that p53 was considered a potential therapeutic target for the treatment of this disease [70].

### 4.4. Possible Current Therapeutic Management of Postmenopausal Osteoporosis

Postmenopausal women and men aged 50 years and older with hip or vertebral fractures, regardless of T-score, should be considered for pharmacological treatment. Fracture incidence goes down after therapy, mostly in patients with previous fractures whether the T-score classification is normal, osteopenia, or osteoporosis. Moreover, in general, no unified advice administers to all patients; management of treatment must be individualized. In accordance with the treat-to-target approach, osteoporotic patients should be risk stratified before starting treatment (Table 2).

Firstly, the goal of medication would be a therapy that succeeds in decreasing the risk in an adequate manner, combining the patient needs [71]. Fracture risk in osteoporotic patients may be lowered by 70% with bone protective therapy; nevertheless, the osteoporotic patients showed a trend of low adherence to treatment of this silent chronic disease. Therefore, it could be useful to establish screening and the best pharmacological and nonpharmacological therapy strategies for reducing fracture [72]. Rehabilitation therapy, such as electrotherapy, kinetotherapy, hydro-kinetotherapy, thermotherapy, psychotherapy, and other programs to avoid falls and ameliorate postural stability, belong to nonpharmacological treatments. These activity plans could enhance the functionality and independence of subjects, improving the quality of life of osteoporotic patients [73]. 

Lifestyle recommendations should include a diet with adequate total calcium intake incorporating calcium supplements if intake is insufficient. Magnesium is essential for calcium absorption, but magnesium extra-supplementation does not increase BMD [74]. De Castro Gomes et al. showed that more than 60% of postmenopausal women with low bone mass took calcium and vitamin D supplements improperly [75]. Excessive chronic intake of vitamin A could have adverse effects on bone. Some data suggest that vitamin K may reduce bone loss in postmenopausal women [74], and it has been detected that constant consumption of soy foods shows a lower risk of osteoporosis than a classic Western diet in these patients [76,77].

Hormone replacement therapy is suggested only for women who have moderate to severe menopause disorders. In current years, research has highlighted the role of phytoestrogens (selective estrogen receptor modulators) as an alternative treatment that can improve the same symptoms but with fewer side effects; among them, soy isoflavonoids have raloxifene-like beneficial effects on the bone metabolism. Many menopausal women use phytoestrogens to preserve their bone mass. Both therapies, the phytoestrogens and hormones, produce a significant decrease in the bone resorption process. The comparative valuation showed no significant differences between the efficacy of the phytoestrogens and the hormone therapy on BMD and bone resorption when administered to groups of women with the same clinical and sociodemographic features [78,79].

## 5. Discussion

### 5.1. Pleiotropic Effect of Physical Activity on Altered Metabolism Age-Related

The results of this study underline that the effects of physical activity spread to multiple levels. It might perform a role in maintaining bone quality by realizing mediators of bones (osteokynes) that influence their expression, acting as intermediaries released by other tissues; or releasing mediators from bone (adipokines, cytokines, neurotransmitters, and myokines) that affect themselves both directly and indirectly and via the specific effect of physical activity on neighboring tissues (skeletal muscle, immune system, adipose system) [80]. Among the myokines, even if irisin was inversely correlated to advancing age, the exact serum levels in elderly people have to be clarified yet [11]. However, these findings suggest that irisin could be a potential marker for staging and early diagnosis of sarcopenia [23]. In the senile population, sarcopenia and OP may share many common pathways, such as the weak reactivity to anabolic molecules secreted by the bone (osteokines) or by the muscle (myokines), the lower hormone production, and also the predisposition to reduced physical exercise [11]. Aging is typically combined with a number of functional and structural changes that may concur to a progressive decline in muscle mass and increase in body fat, and a related decrease in BMSCs differentiation to osteogenic line cells could be induced by an imbalance in adipo-osteogenic differentiation [35]. BMSCs are the common source that binds trabecular bone, bone marrow fat, osteoblasts, and adipocytes; moreover, their differentiation into osteoblasts or adipocytes could be influenced by local factors and hormones [19].

Physical exercise is considered the main negative factor regulator of age-related mutations and could perform a positive anabolic effect on bone either indirectly through endocrine regulation or directly via mechanical signals caused by muscle contraction, supporting the close interaction between bone and muscle [35]. One of the main endocrine organs that could control energy homeostasis and imbalance is the adipose tissue; the functions of WAT and BAT are different and, respectively, have been classified as storage of energy in the form of triglycerides, while BAT is intricate in the purpose of insulin sensitivity, non-shivering thermogenesis, and energy dissipation. Some adipocytes from the storage of WAT, after any stimulation, have the ability to change, gaining characteristics of BAT. The activation of BAT or induction of WAT browning has shown that it could also prevent bone loss-associated diseases [3]. The evidence that a positive connection between lean body mass and bone mineral content endures for the entire life validates the existence of this functional unit. Furthermore, a gain in muscle strength anticipates an increase in bone strength [2].

Age-related alterations could involve not only tissue loss but also metabolic and endocrine deviation linked to an elevation of proinflammatory cytokines [35]. The basal level of circulating irisin was significantly lower in patients with metabolic syndrome and previous osteoporotic fractures than in healthy centenarians [81]. Irisin, generated in response to physical activity, is noted to be valid to induce an increment of energy expenditure of WAT and consequently define a body fat reduction [3]. Therefore, it has been found useful as a potential treatment for metabolic disorders; in addition, other data also described the prospective functions of this myokine in the promotion of bone metabolism by regulating dynamic osteoblast balance [10]. Planned physical activity could finely control bone metabolism; indeed, moderate intensity and long duration could raise bone mass and decrease bone resorption in both pathological and healthy people [1], and it could delay the onset of OP through the improvement in peak bone mass during youth [82].

### 5.2. Influence of Different Types of Physical Activity

Little evidence is available regarding which type of exercise session elicits a more strong effect on the release of myokines or proteins/hormones. Further, it is important to know the existence of wide inter-individual variability in the biological responses to a given exercise session to ensure a better-personalized exercise prescription. In fact, there are some subjects achieving meaningful benefits (known as “responders”) and others showing no changes (“non-responders”) [83]. Exercise is classified into six categories: high-impact weight-bearing exercise such as dancing or running, low-impact weight-bearing exercise such as walking, static weight-bearing exercises such as single-leg balance, low-impact non-weight-bearing exercises such as swimming, high-impact non-weight-bearing exercise, and combination of exercise [84].

A significant increase in irisin levels during an acute bout of exercise to exhaustion has been observed immediately after high-intensity interval exercise (HIIE), continuous moderate-intensity exercise (CME), and resistance exercise (RE) and declined 1 h later, indifferently in individuals with metabolic syndrome as well as healthy individuals [85]. HIIT, which involves short repeated bursts of powerful exercise (from less than 1 min to a maximum of 2–4 min) interspersed with short rest time, is gaining considerable popularity, partly owing to the short period commitment it requires [83]. In addition, recent research has demonstrated the beneficial effects of HIIT also in individuals with metabolic disease [38]. It has been shown that high-intensity concurrent exercise after 10 weeks, compared to the moderate-intensity continuous concurrent exercise (MCC) group, has a greater influence on reducing metabolic syndrome in women of postmenopausal age, improving most metabolic-related parameters than moderate-intensity concurrent exercise [86]. Endurance exercise has been reported to be associated with important metabolic mediators. A meta-analysis study showed that peripheral irisin levels may be increased immediately after an acute bout of exercise in young and middle-aged people [87]. Aerobic program exercise ameliorated postural performance in postmenopausal elderly women; indeed, among the most common causes of falls that have been investigated are balance disorders and gait caused by impaired general health status and consequent decreased postural stability [48]. In 2018, it was also found that a high-intensity jump-based aquatic exercise program was efficient in improving bone mass and functional fitness in postmenopausal women [88]. Another example of a safe and high-energy exercise is considered dance, reporting a positive effect also on femoral neck BMD but not in spine BMD, probably caused by a better effect to the trabecular bone than to cancellous bone. Muscles increase in size and power after aerobic exercise therefore using a high volume of low-intensity muscular contractions it improves side step and grip strength, an indicator of likely future disabilities and functional limitations. Meanwhile, the regular exercise program facilitated neuromuscular control of the body, so reaction time was better as well. Therefore differently from other studies about exercise regimen 12 months of high-impact [89] is possible that also a 24-week aerobic dance program could reduce the incidence of falls in postmenopausal women improving femoral neck BMD [90] (Table 3).

Irisin levels, according to the diurnal change concentration over 12 h, are related to the type of physical activity usually performed and to muscular strength, contractility and volume. During physical activity, a better increase in irisin concentration has been shown after anaerobic activity compared to aerobic activity; the levels of irisin proved better in resistance exercise compared with endurance exercise alone and resistance and endurance exercises combined. Moreover, a study in adults reported an increase in irisin following acute exercise but a decrease in irisin after a 12-week period of endurance and strength training. Irisin levels are a determinant of measures of areal and volumetric bone mineral density and bone strength estimates in athletes [91]. Even high-volume resistance exercises on the whole body led to the gain in the concentration of irisin in the 1 h after exercise differently from the exercise performed on a single muscle district. Furthermore, an increase in the concentration of irisin has also been detected after vibration exercise [92]. The exercise length could play a key role in bone setting even if endurance training and high-intensity resistance it is applied [93]. These findings showed that strength exercise can modulate biomechanical parameters of bone tissue and bone micromilieu during senescent and effects of different program exercise could modify the differential expression of some miRNAs, identified as a controller in bone homeostasis targeting bone resorption factor and osteogenic factors, such as the transcript factor of adipogenesis or osteogenesis [12].

Emerging evidence reported that muscle-bone communication may be obtained by the release of myotube-derived exosomal miRNAs, called myomiRs, and some osteocyte-derived exosomal micro(mi)RNAs, called osteomiRs, to their microenvironment cells. So, myokines and myomirs can also be mediators of intracellular pathways between neighbor and far tissue [94]. The transcriptional program required for an optimal differentiation among osteoblast lineage could involve complex pathways regulated both at the transcriptional and post-transcriptional grades and activated by distinct second messenger pathways, which are not yet fully investigated [51]. The literature shows that OP is linked to an altered expression of circulating miRNAs stems detected by microarray analysis of 365 miRNAs in human circulating monocytes gathered from postmenopausal Caucasian women with either low or high BMD, finding miR-133 significantly upregulated in the low-BMD patients compete with their normal BMD model [13]. Other authors showed an upregulation of miR-133 in the OP and osteopenia women compared with the controls, and both correlated with low BMD values [95]. Other studies correlate tissue and circulating miRNAs expression with the risk of bone fracture in senile OP patients. MiR-214 expression has been related negatively to bone formation markers levels and positively with age [56]. Yet, in 2018, Sansoni et al. demonstrated that some fracture risk-associated miRNAs responded to a protocol of physical activity in a more sensitive manner than cytokines, standard bone metabolism markers, and metabolic hormones [96].

Different types of exercise could stimulate bone formation, and the literature has already shown that these effects could have a beneficial rebound on BMD in postmenopausal women [97]. Physical activity could both regulate production of irisin and consequently reduce the synthesis in skeletal muscle of myostatin in an inverse manner, leading to an increase in mineral content and bone cortical and trabecular area [27] and may also reduce bone sclerostin production, causing, subsequently, a decrease in bone loss via osteoclastogenesis and enhancement of osteoblastic bone formation [30].

## 6. Conclusions and Future Perspectives

Recently the regulatory functions of epigenetic factors, such as miRNAs, in the physiological and pathological processes have been widely investigated. In particular, modulation of miRNAs following exercises represents an interesting field of investigation since these non-coding RNAs may be regarded as useful prognostic markers, as well as a protector against disease. Moreover, their rapid yet transient regulation with exercise prompts that they may concur to the positive adaptation, modulating various intracellular stress signals by targeting, or triggering, the expression of master genes coding for specific transcription factors that may control the mutually exclusive fate of progenitors cells.

Based on this integrated perspective, this narrative review may represent a relevant help to future investigation aimed at characterizing the possible role of specific types of physical activity to condition miRNAs expression and, consequently, their involvement in the pathways related to chronic bone disease, such as OP. Therefore, it would be useful to find the potential ability of those treatments, both pharmacological and alternative, such as physical exercise, to modify transcription factors by acting on an epigenetic level.

It would be helpful to investigate how exercise could facilitate osteoinduction; indeed, physical activity has been proposed as a supplementary method of recovering bone health in regenerative medicine.

Mechanical load, while stimulating irisin, downregulates myostatin levels, linking the functional bone-muscle unit. Therefore, the emerging role of irisin and its low levels in postmenopausal women with sarcopenia status suggested that irisin could be a potential marker for staging and early diagnosis for sarcopenia. Moreover, the treatment with a low dose of recombinant irisin was able to gain in quality of bone mass; therefore, it would be useful to highlight its role as a therapeutic target in osteoporotic aging in the future implications. Further studies need to verify if the different impacts on various transcription factors could be conditioned by different types of exercise. With a view to the future, supportive care, such as regular physical activity, acting on the miRNAs balance adjusting their transcription factors involved in different chronic disorders, could decrease the pharmacological load and would be able to help modify the clinical history of the disease.

## Figures and Tables

**Table 2:** Possible management of postmenopausal osteoporosis according to fracture risk.

Stratification of Risk	Low Risk (No Previous Spine or Hip Fracture; T-Score at Hip and Spine above −1.0; Normal Bmd)	Moderate Risk (No Previous Spine or Hip Fracture; T-Score between −1.0 and −2.5; Low Bone Mass)	High Risk (Prior Spine or Hip Fracture; or a Lumbar Spine or Hip T-Score of −2.5 or Below; Osteoporosis)	Very High Risk (Multiple Spine Fractures/Hip Fracture and T-Score of −2.5 or Lower at Lumbar Spine or Hip; Severe or Established Osteoporosis)
**Non** **Pharmacological** **Treatments**	Optimize Calcium and Vitamin D Status			
	Risk Appropriate Exercise		Risk Appropriate Exercise and Falls Preventions	
	Lifestyle recommendation	Weight-bearing activities; Muscle-strengthening activities; Balance, posture, and functional activities.		
**Pharmacological** **Treatments**	Not recommended	Consider FDA-approved medical therapies in adults with low bone mass (osteopenia) and with a 10-year probability of a hip fracture ≥ 3% or 10-year probability of any major osteoporosis-related fracture ≥ 20%.	Initial treatment with inhibitors of bone resorption Bisphosphonates. Denosumab (as alternative therapy to reduce fracture risk.) To reduce the risk of vertebral fractures in postmenopausal women with a low risk for deep vein thrombosis and for whom Bisphosphonates or Denosumab are not appropriate: Estrogen related therapy In women with a high risk of breast cancer treatment recommended preventing vertebral fractures is: Raloxifene or Bazedoxifene	Initial treatment with stimulators of bone formation. Teriparatide or Abaloparatide treatment for up to 2 years or Romosozumab for 1 year. Following a course of anabolic treatment with antiresorptive osteoporosis therapies should be used to maintain bone density gains.
**Follow-up**		Regularly assess compliance and persistence with the therapeutic regimen (at least annually)		
	Reassess Fracture Risk in 2 to 4 Years	Reassess Fracture Risk in 2 to 4 Years		

**Table 3:** Impact of different exercises on bone metabolism in osteoporosis (OP).

Reference Year Author	Article Type	Population	Duration and Type of Exercise	Effects
[32] 2017 Singulani, M.P et al.	Article	Adult aged vs. exercised aged rats	-ST -16 weeks	ST: -Increase in: biomechanical parameters, runx2, osx gene, and bone matrix protein expression -Reduction in: pparγ expression and risk of fractures during senescent
[42] 2019 Gonzalo-Encabo, P. et al.	Clinical trial study	400 idle postmenopausal woman	High rates vs. moderate rates of aerobic exercise -12 month	HIGH RATES OF AEROBIC EXERCISE: -Improves BMD -Attenuates age-related declines Benefits persist at intervention concludes
[43] 2017 Zhao, R. et al.	Review	1061 postmenopausal women from 11 RCTs	-Combined exercise -Data at 12 month	Increase in BMD Prevents postmenopausal bone loss and helps in reducing risk of fracture
[44] 2015 Ju, Y. I. et al. [45] 2016 Oh, T. et al.	Articles	OVX rats	-Swimming -12 weeks -High-strength intermittent swimming training -6 weeks	-Trabecular architectural changes in cancellous bone -Beneficial effect on bone quantity and intensity
[46] 2016 Pereira, A. et al.	Article	11 postmenopausal women	-Combined training -16 week	Improves dynamic muscular strength in lower and upper limbs No change in bone resorption
[47] 2013 Mosti, M.P. et al.	Article	21 postmenopausal women with OP or osteopenia	-Squat exercise -12 weeks	MAXIMAL STRENGTH TRAINING: Improves BMD and bone mineral content
[48] 2008 Gunendi, Z. et al.	Article	25 postmenopausal women without OP vs. 28 postmenopausal women with OP	-Submaximal aerobic exercise program on treadmill -Twice a week for 4 weeks	Improves static and dynamic postural balance in postmenopausal women with OP No changes in BMD
[49] 2020 Mittlmeier, T.	Article	OVX rats	Low-intensity endurance training on the treadmill vs. medium and HIIT -5 week	TREADMILL TRAINING: Influences differentially on the musculoskeletal unit
[57] 2021 Kawao, N.	Article	OVX mice	-Treadmill exercise with moderate intensity -8 week	Irisin high level might be related to increases in trabecular BMD but not with cortical BMD improvement in decreases in trabecular and cortical BMD
[67] 2019 Kim Jeong-Seok	Article	OVX rats	-Interval running -6 week	Decreases bone resorption prevents skeletal muscle wasting and bone loss in the elderly
[88] 2018 Aboarrage Junior, A.M. et al.	Article	25 postmenopausal women	-High-intensity jump-based aquatic exercise program (HIIAE)	Improves BMD
[89] 2005 Vainionpää, A. et al.	Article	120 premenopausal women	-Progressive high-impact exercises -3 times per week for 12 months	Improves BMD prevents OP
[90] 2019 Yu, P. et al.	Article	100 postmenopausal women	-Aerobic dancing -24 weeks	Improves: BMD, muscle strength, agility. High-impact exercise better influence the trabecular bone than cancellous bone

ST: strength training; Runx2: runt-related transcription factor 2; osx: osterix; pparγ: proliferator-activated receptor gamma, BMD: bone mineral density; vs.: versus; RCTs: randomized controlled trials; OVX: ovariectomized; OP: osteoporosis; HIIT: high-intensity interval training; HIIAE: high-intensity jump-based aquatic exercise program.

## Data Availability

Not applicable here.
